# Integrative metabolome and genome-wide transcriptome analyses reveal the regulatory network for bioactive compound biosynthesis in lettuce upon UV-A radiation

**DOI:** 10.1186/s43897-025-00163-1

**Published:** 2025-08-05

**Authors:** Lingyan Zha, Shiwei Wei, Xiao Yang, Qingliang Niu, Danfeng Huang, Jingjin Zhang

**Affiliations:** 1 School of Agriculture and Biology, Shanghai Jiao Tong University, Shanghai, 200240 China; 2 Shanghai Agrobiological Gene Center, Shanghai, 201106 China; 3 Institute of Urban Agriculture, Chinese Academy of Agricultural Sciences, Chengdu National Agricultural Science and Technology Center, Chengdu, 610213 China

**Keywords:** Phenylpropanoid, Vitamin, Sesquiterpenoid, Transcriptional regulation, MYB, WRKY

## Abstract

Ultraviolet A (UV-A) radiation possesses great potential for enhancing the bioactive properties of vegetables and also has promising application prospects in controlled-environment agriculture. Lettuce is a widely cultivated model vegetable in controlled-environment agriculture with abundant health-beneficial bioactive compounds. However, the comprehensive regulatory effectiveness and mechanism of UV-A on bioactive compounds in lettuce remain largely unclear. To address this issue, we performed transcriptomic and metabolomic analyses of UV-A-treated lettuce to construct a global map of metabolic features and transcriptional regulatory networks for all major bioactive compounds. Our study revealed that UV-A promotes the accumulation of most phenylpropanoids and vitamins (provitamin A and vitamin E/K_1_/B_6_) but represses the biosynthesis of sesquiterpenoids. MYB transcription factors (TFs) are key activators of bioactive compound biosynthesis promoted by UV-A, whereas WRKY TFs primarily inhibit the production of sesquiterpenoids. Moreover, light signaling plays a crucial and direct regulatory function in stimulating the biosynthesis of phenylpropanoids and vitamins but not in that of sesquiterpenoids. In comparison, hormone signaling dominates a more decisive regulatory role in repressing sesquiterpenoid biosynthesis through working directly and interacting with WRKY TFs. This study paves the way toward an understanding of the bioactive compound regulation and genetic improvement of lettuce bioactivity value.

**Supplementary Information:**

The online version contains supplementary material available at 10.1186/s43897-025-00163-1.

## Core

UV-A promotes the accumulation of most phenylpropanoids and vitamins (provitamin A and vitamin E/K_1_/B_6_) but represses the biosynthesis of sesquiterpenoids in lettuce. MYB and WRKY transcriptional factors serve as master regulators in regulating the biosynthesis of UV-A-promoted and UV-A-repressed bioactive compounds, respectively. Light signaling plays a crucial and direct regulatory role in modulating the biosynthesis of phenylpropanoids and vitamins but not in that of sesquiterpenoids, which is more closely correlated with hormone signaling.

## Gene and accession numbers

The sequence data are available in the form of fastq files in SRA NCBI database (https://www.ncbi.nlm.nih.gov/sra/) under accession No. PRJNA916618

## Introduction

Currently, consumers’ requirements for food are not only confined to nutritional needs but also concentrated more on health-promoting and disease-prevention effects, which are thought to be closely linked to the bioactive compounds in foods (Septembre-Malaterre et al., 2018). Lettuce (*Lactuca sativa* L.) is one of the most widely consumed vegetables worldwide, with abundant minerals and various health-beneficial bioactive compounds (Kim et al., 2016). Since lettuce is typically eaten raw, it avoids the inactivation of bioactive compounds during cooking and processing, making lettuce an ideal dietary source of health-benefiting bioactive compounds. The most abundant components of health-promoting bioactive compounds in lettuce include hydroxycinnamic acids, glycosylated flavonoids, carotenoids (provitamin A), tocopherols (vitamin E), ascorbic acid (vitamin C), group B vitamins, and sesquiterpene lactones; these components greatly contribute to the anti-inflammatory, cholesterol-lowering, and anti-diabetic activities of lettuce (Kim et al., 2016; Yang et al. 2022a). On the basis of their compound classification and biosynthetic origins, these bioactive compounds in lettuce can be roughly categorized into phenylpropanoids (phenolic acids, flavonoids, and anthocyanins (purple leafy lettuce)), lipid-soluble vitamins (vitamins A, E, and K), water-soluble vitamins (vitamins B and C), and sesquiterpenoids, which are derived from the phenylpropanoid pathway, methylerythritol phosphate pathway, carbohydrate metabolism, and mevalonate pathway, respectively.

In terms of cultivation, lettuce also serves as the most widely cultivated model vegetable in controlled-environment agriculture, particularly in plant factories with artificial lighting (PFALs), owing to its favorable agronomic traits and excellent suitability for hydroponic production (Kozai et al. 2020). Although the productivity and food safety of lettuce cultivated in controlled-environment cultivation systems significantly surpass those of open-field cultivation, its nutritional quality, particularly bioactive compound levels, tends to be much lower. It has been noted that ultraviolet (UV) deficiency is one of the main issues responsible for the scarcity of bioactive compounds in vegetables cultivated in horticultural facilities (Neugarta and Schreiner, 2018), as the accumulation of numerous bioactive compounds is primarily associated with bolstering plant resistance to environmental stimuli, including UV radiation (Wargent and Jordan 2013; Yang et al. 2022a). To date, available UV-relevant studies predominantly focused on UV-B, which has been extensively proven to promote the accumulation of various health-promoting compounds but brings a detrimental effect on plant growth (Neugarta and Schreiner, 2018; Wargent and Jordan 2013). In comparison, UV-A has a less harmful influence and is safer for both plants and humans than UV-B. Moreover, UV-A can reach deeper leaf tissues with stronger penetration, which contributes to the accumulation of bioactive compounds (Neugarta and Schreiner, 2018; Verdaguer et al., 2017). Recent reviews and studies have reported that many bioactive compounds, such as phenolic acids, flavonoids, vitamin C, and glucosinolates, exhibit greater levels in plants exposed to UV-A radiation (Jiang et al. 2023; Lauria et al. 2024). Additionally, with recent advancements in UV-emitting LED technology, the price of UV-A LEDs is merely 10% that of UV-B LEDs. Consequently, when holistically considering safety, efficacy, and cost-effectiveness, UV-A has emerged as a potentially optimal light quality for enhancing the bioactive compound level in vegetables cultivated in controlled-environment agriculture.

Although UV-A radiation has considerable potential for promoting the bioactive value of vegetables and their application in controlled-environment agriculture, a comprehensive understanding of UV-A regulation of bioactive compounds and the corresponding regulatory mechanisms is still lacking. Multiomics technology can be used to assess systematic changes in metabolites and genes comprehensively, making it a powerful approach for addressing this knowledge gap. To date, omics analyses have been widely employed to decipher the metabolic features and relevant regulatory factors of numerous health-benefiting metabolites, particularly in important economic vegetables, fruits, and grain crops, such as tomato (Li et al. 2020), kiwifruit (Shu et al. 2023), apple (Li et al. 2024), and rice (Yang et al. 2022b). In the case of lettuce, recent studies based on multiomics analysis have clarified the phytochemical accumulation pattern and coordinated transcriptional regulation in response to light quality (Kitazaki et al. 2018; Yamashita et al. 2023), cold stress (Yang et al., 2024), and nitrogen (Liang et al., 2023). Coincidentally, all these studies focused on flavonoids but lacked in-depth analysis and exploration of other phytochemical compounds. Thus, further dissection of the metabolic features and metabolic regulatory networks of prominent bioactive compounds in lettuce needs to be pushed forward, which is a prerequisite for the comprehensive and accurate improvement of nutritional quality in lettuce.

In this study, we aimed to investigate the accumulation features of bioactive compounds in lettuce under UV-A exposure and their underlying regulatory mechanisms. Based on metabolomic and transcriptomic profiling of UV-A-treated lettuce leaves, we systematically characterized UV-A-induced changes in bioactive compound accumulation at both metabolic and transcriptional levels. Subsequently, by integrating weighted gene coexpression network analysis (WGCNA), Pearson correlation coefficient analysis, and motif-based sequence analysis, we established metabolic regulatory networks for UV-A-regulated bioactive compounds to screen potential transcription factors (TFs) that directly bind structural genes governing the biosynthesis of these compounds. In addition, we further explored the regulatory role of light and hormone signaling in modulating the synthesis of different bioactive compounds. Overall, our study provides a comprehensive framework for understanding the response of distinct bioactive compounds to UV-A radiation and offers guidelines for manipulating and improving the bioactivity of lettuce.

## Results

### The comprehensive effect of UV-A on the accumulation of bioactive compounds in lettuce at the metabolic and transcriptional levels

**Fig. 1:**
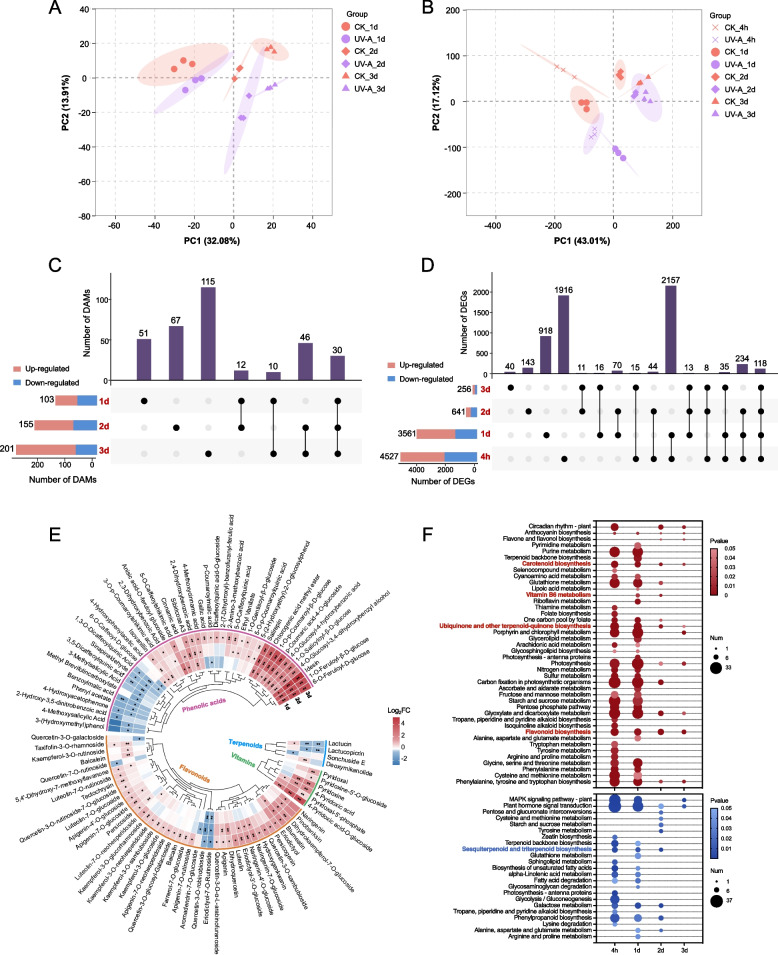
Effects of UV-A on the accumulation of bioactive compounds in lettuce at metabolic and transcriptional levels. **A**,** B** Principal component analysis for metabolomes (**A**) and transcriptomes (**B**). **C**, **D** UpSet plots of differentially accumulated metabolites (DAMs, VIP ≥ 1 and *p*-values ≤ 0.05) (**C**) and differentially expressed genes (DEGs, *p*-adjust ≤ 0.05 and |log_2_FC|≥ 0.58) (**D**) in comparison group of UV-A vs CK. **E** Heatmap illustrating the Log_2_FC of DAMs belonging to bioactive compounds in the comparison group of UV-A vs CK. The * and ** indicate *p*-values ≤ 0.05 and *p*-values ≤ 0.01 respectively. **F** KEGG enrichment analysis of up/down-regulated (red/blue) DEGs (*p*-adjust ≤ 0.05 and |log_2_FC|≥ 0.58) in the comparison group of UV-A vs CK

To gain comprehensive insight into the response of bioactive compounds to UV-A radiation, metabolic and transcriptomic analyses were conducted to monitor metabolic changes and related biosynthetic pathways at the transcriptional level. Principal component analysis (PCA) of the metabolomes and transcriptomes revealed high repeatability among the biological replicates at each time point (Fig. 1A, B). By analyzing the differentially accumulated metabolites (DAMs, UV-A vs CK, VIP ≥ 1 and *p* values ≤ 0.05) and differentially expressed genes (DEGs, UV-A vs CK, *p*-adjust ≤ 0.05 and |log_2_FC|≥ 0.58) between the UV-A and control treatments at different time points, we found that the number of DAMs increased with increasing treatment time (Fig. 1C), but the number of DEGs decreased with treatment time and sharply decreased at 2 d (Fig. 1D). The relative contents of DAMs belonging to phenolic compounds, vitamins, and terpenoids were further analyzed to evaluate the effects of UV-A on bioactive compounds. A total of 42 phenolic acids, 41 flavonoids, 6 vitamins, and 4 sesquiterpenoids were screened by further employing |log_2_FC|≥ 0.58 as the threshold (Fig. 1E). Specifically, for phenylpropanoids in lettuce, the majority of phenolic acids and almost all flavonoids exhibited significantly higher levels in UV-A leaves, and those elevated phenolic acids presented greater and faster responses to UV-A radiation compared with flavonoids. Vitamins with log_2_FC values greater than 0.58 all belong to the vitamin B6 group, among which 4-pyridoxic acid-O-glucoside was notably elevated 6- to 20-fold by UV-A. However, for terpenoids, sesquiterpenoids, a class of bioactive substances unique to Asteraceae plants, were significantly down-regulated by UV-A (Fig. 1E).

To reveal metabolite accumulation from a genetic basis, we performed Kyoto Encyclopedia of Genes and Genomes (KEGG) enrichment analysis on the up- and down-regulated DEGs at each time point. Consistent with the metabolites, significant enrichment of upregulated DEGs in the ‘flavonoid biosynthesis’ and ‘vitamin B6 metabolism’ pathways, as well as enrichment of down-regulated DEGs in the ‘sesquiterpenoid and triterpenoid biosynthesis’ pathway, were observed (Fig. 1F). In addition, the upregulated DEGs were consistently and remarkably enriched in pathways that are responsible for the synthesis of lipid-soluble vitamins (carotenoids (provitamin A), tocopherols (vitamin E), and phylloquinone (vitamin K1)), i.e., the ‘carotenoid biosynthesis’ and ‘ubiquinone and other terpenoid-quinone biosynthesis’ pathways. Therefore, we obtained the contents of carotenoids and chlorophyll via chemical methods to provide a rough reference of lipid-soluble vitamin contents, as a significant positive correlation was found between the contents of chlorophyll and phylloquinone, with a ratio of 9 mmol/mol (phylloquinone/chlorophyll) (Kodaka et al. 1986). As expected, UV-A-treated leaves had significantly higher carotenoid and chlorophyll contents, as well as a greener phenotype than control leaves (Fig. S1). Moreover, although the upregulated DEGs were also considerably enriched in pathways related to the synthesis of several other water-soluble vitamins (‘riboflavin metabolism’, ‘thiamine metabolism’, ‘folate biosynthesis’, and ‘ascorbate and aldarate metabolism’) at 4 h or 1 d (Fig. 1F), the relative contents of folate, riboflavin, and dehydroascorbic acid were greater in UV-A leaves only when VIP ≥ 1 was considered as the screening threshold (Table S1). Thus, combining metabolome and transcriptome data, we conclude that UV-A can effectively promote the accumulation of the majority of bioactive compounds in lettuce, including phenolic acids, flavonoids, and vitamins (B_6_, E, K_1_, and provitamin A), but inhibit the synthesis of sesquiterpenoids.

### Construction of coexpressed gene networks and transcription factors identification

**Fig. 2:**
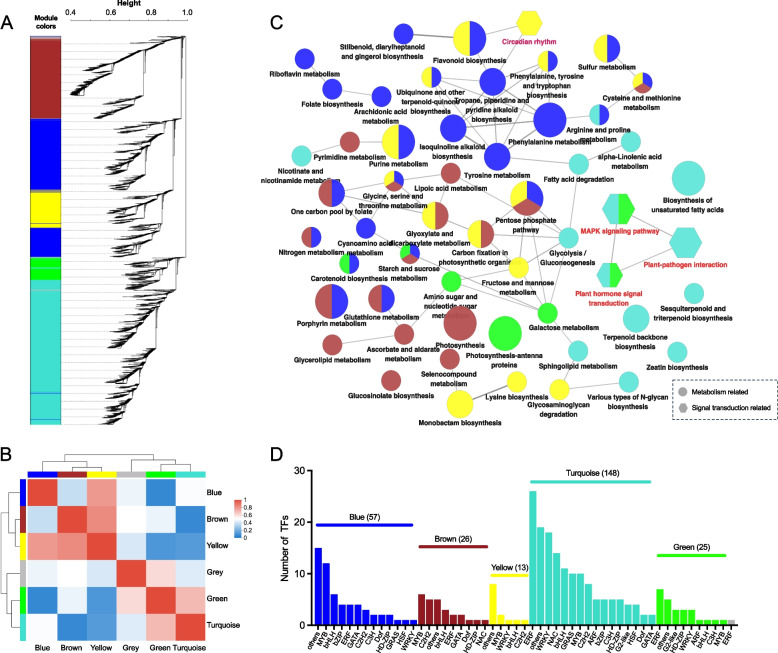
Weighted gene co-expression network analysis of total DEGs (UV-A vs CK, *p*-adjust ≤ 0.05, and |log2FC|≥ 0.58). **A** Clustering dendrogram of genes and module division. Each leaf represents a single gene, 6 modules were defined and coded by different colors. **B** Correlation between modules. **C** KEGG enrichment analysis of genes in each module. Pathways with *p* ≤ 0.05 were visualized by ClueGO of Cytoscape. The symbol size indicates the *p*-value, the smaller the *p*-value, the larger the size. Lines between symbols indicate the existence of connections between metabolic pathways. The color of symbols corresponds to the module color. **D** Number of TF family members in each module. The color of the columns corresponds to the module color

To gain further insight into the regulatory mechanism of UV-A on bioactive compounds in lettuce, WGCNA was performed with a collection of DEGs identified at each time point. A total of 6 coexpression modules were identified on the basis of their similar expression patterns (Fig. 2A; Table S2). Analysis of the module − module and module − trait correlations revealed that highly positive correlations existed between the blue, brown, and yellow modules and between the turquoise and green modules (Fig. 2B). The former three modules were positively correlated with certain samples subjected to UV-A treatment, but the last two modules were negatively correlated with certain UV-A samples (Fig. S2). KEGG enrichment analysis of different modules revealed that genes involved in ‘flavonoid biosynthesis’, ‘carotenoid biosynthesis’, and ‘ubiquinone and other terpenoid-quinone biosynthesis’ pathways were enriched mainly in the blue and/or yellow module, whereas most of the genes related to the “terpenoid backbone biosynthesis” (mainly the MVA pathway) and “sesquiterpenoid and triterpenoid biosynthesis” were located in the turquoise module (Fig. 2C). Additionally, the “circadian rhythm” pathway was found to be significantly enriched in the yellow module, whereas the “plant hormone signal transduction” and “MAPK signaling” pathways displayed notable enrichment in the turquoise module. In addition, based on the PlantTFDB database, we identified a total of 270 TFs within the collection of DEGs, which were categorized into 36 TF families, including ERF (40), MYB (31), WRKY (23), bHLH (22), C2H2 (17), NAC (15), GRAS (11), HD-ZIP (11), bZIP (9), C3H (8), GATA (8), G2-like (7), and others. Notably, MYBs constituted the largest TF family in all the blue, brown, and yellow modules, while the ERF and WRKY families were coexpressed mainly in the turquoise and green modules (Fig. 2D). These findings suggest that light signals and MYB TFs are likely involved in the regulation of the biosynthesis of phenolic acids, flavonoids, and vitamins, whereas hormonal signals, along with ERF and WRKY TFs, have significant potential in modulating the biosynthesis of sesquiterpenoids.

### Identification of key transcription factors in regulating phenylpropanoid biosynthesis upon UV-A radiation

**Fig. 3:**
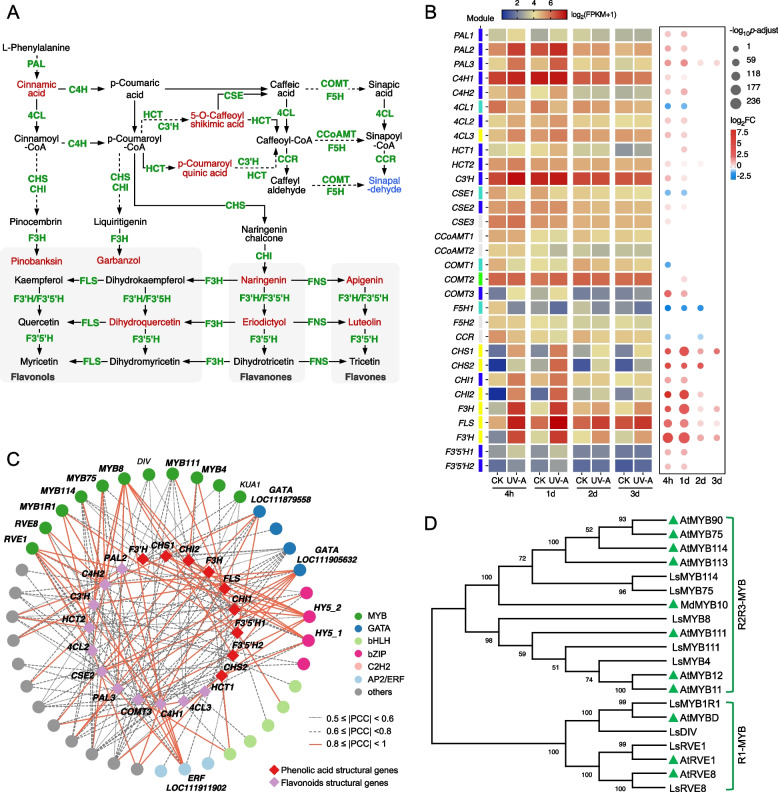
Comprehensive analysis and regulatory network of genes involved in phenylpropanoid biosynthesis in lettuce under UV-A. **A** Schematic of phenylpropanoid biosynthesis pathways. Compounds up-/down-regulated by UV-A are shown in red/blue fonts, respectively. **B** Heat map of the genes encoding structural enzymes involved in the phenylpropanoid biosynthesis pathways. The scaled values of FPKM and Log_2_FC were presented. **C** Regulatory network connecting predicted TFs and key structural genes had potential binding affinity (FIMO match *p*-value ≤ 1 e^−4^) and significant Pearson’s correlation (*p* ≤ 0.01). Expression correlations between TFs and structural genes are shown with line color and type. The full data set is available in Supplementary Table S3. **D** Phylogenetic tree of candidate MYB proteins with others related to phenylpropanoid biosynthesis. The known MYB proteins from other plants were remarked with green triangles. The accession numbers of protein sequences are available in Table S4

Phenylpropanoids are the most abundant phytochemicals in lettuce and are categorized into various subgroups, including phenolic acids, flavonoids, and anthocyanins. Notable and widespread increases in most phenolic acids and flavonoids were observed following UV-A treatment. To clarify the mechanism underlying the regulation of phenylpropanoid biosynthesis by UV-A, the transcription features of genes responsible for the common biosynthesis steps for phenolic acids and flavonoids were analyzed (Fig. 3A, B). The expression levels of genes encoding phenylalanine ammonia-lyase (PAL), cinnamate 4-hydroxylase (C4H), and 4-coumaroyl CoA ligase (4CL)—key enzymes constituting the backbone of the phenylpropanoid pathway—were observed to be 1.7- to 3.7-fold greater in UV-A-treated leaves than in control leaves after 4 h. In comparison, genes related to flavonoid biosynthesis presented much greater responses to UV-A, including *CHS* (chalcone synthase), *CHI* (chalcone isomerase), *F3H* (flavanone 3-hydroxylase), *FLS* (flavonol synthase), *F3′H* (flavonoid 3′‐hydroxylase), and *F3′5′H* (flavonoid 3',5'-hydroxylase), whose transcription levels surged to 3- to 400-fold greater in UV-A leaves than in control leaves at 4 h. In alignment with the metabolite accumulation patterns of UV-A-reduced phenolic acids, such as sinapinaldehyde, several isoforms of caffeic acid 3-O-methyltransferase (COMT), ferulate-5-hydroxylase (F5H), cinnamoyl-CoA reductase (CCR), and caffeoyl CoA 3‐O‐methyltransferase (CCoAMT) exhibited lower or unchanged transcription levels in UV-A-treated leaves. Given that the upregulated genes related to phenylpropanoid biosynthesis were predominantly clustered in the blue and yellow modules, and considering the strong correlations among the blue, brown, and yellow modules, we constructed a regulatory network of TFs and phenylpropanoid structural genes from these three modules to identify potential TFs regulating phenylpropanoids synthesis (Fig. 3C). A total of 24 candidate TFs were identified by integrating both the potential binding affinities and Pearson’s correlation coefficients between the TFs and structural genes (FIMO match *p* value ≤ 1 e^−4^, |PCC|≥ 0.8). Among them, the MYB (8), GATA (2), bHLH (2), and bZIP (2) families emerging as the top four putative TF families engaged in the regulation of phenylpropanoid biosynthesis by UV-A. Previous studies have underscored the pivotal roles of the MYB family in phenylpropanoid synthesis (Cao et al. 2024). Hence, we employed the identified MYB TFs alongside MYB members known to be involved in phenylpropanoid synthesis to construct a phylogenetic evolutionary tree (Fig. 3D). As shown within the tree, those candidate MYB TFs mainly belong to R2R3-MYB and R1-MYB subfamily. Among them, LsMYB4/8/111 formed a cluster with AtMYB11/12/111, LsMYB75/114 clustered with AtMYB75/90/113/114 and MdMYB10, and R1-MYBs in lettuce have strong homology to those in Arabidopsis.

### Identification of key transcription factors in regulating vitamin biosynthesis upon UV-A radiation

**Fig. 4:**
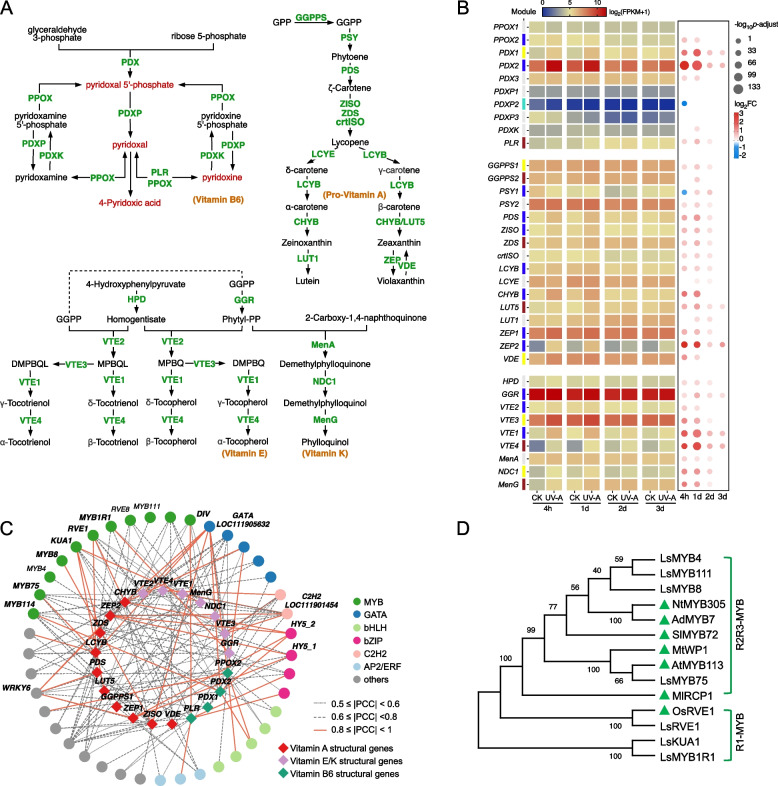
Comprehensive analysis and regulatory network of genes involved in vitamin biosynthesis in lettuce under UV-A. **A** Schematic of vitamin biosynthesis pathways. Compounds up-regulated by UV-A are shown in red. **B** Heat map of the genes encoding structural enzymes involved in the vitamin biosynthesis pathways. The scaled values of FPKM and Log_2_FC were presented. **C** Regulatory network connecting predicted TFs and key structural genes had potential binding affinity (FIMO match *p*-value ≤ 1e.^−4^) and significant Pearson’s correlation (*p* ≤ 0.01). Expression correlations between TFs and structural genes are shown with line color and type. The full data set is available in Supplementary Table S3. **D** Phylogenetic tree of candidate MYB proteins with others related to vitamin biosynthesis. The known MYB proteins from other plants were remarked with green triangles. The accession numbers of protein sequences are available in Table S4

The markedly increased levels of vitamin B_6_ group compounds and carotenoid content (Fig. 1, S1), as well as the significant enrichment of upregulated DEGs in the “vitamin B_6_ metabolism”, “carotenoid biosynthesis”, and “ubiquinone and other terpenoid-quinone biosynthesis” pathways, indicate that UV-A can activate the biosynthesis of several vitamins, including vitamins B_6_, E, and K_1_ and provitamin A. To clarify the transcriptional regulatory mechanism of UV-A on these vitamins, we analyzed the expression patterns of structural genes implicated in the biosynthesis pathways of these vitamins. By incorporating insights from the KEGG database, we annotated 10, 16, and 9 structural genes involved in the vitamin B_6_, provitamin A, and vitamin E/K_1_ biosynthesis steps, respectively (Fig. 4A, B). Almost all biosynthetic genes for these vitamins presented significantly greater expression levels in UV-A-treated leaves. Among them, *CHYB* (β-carotene hydroxylase), *LUT5* (β-ring hydroxylase), *ZEP* (Zeaxanthin epoxidase), *VTE1* (Tocopherol cyclase), and *VTE4* (Tocopherol O-methyltransferase), whose coding products catalyze the synthesis of β-carotene, zeaxanthin, and α-tocopherol expressed stronger responses to UV-A radiation. With respect to vitamin B_6_, although UV-A exerted little influence on the expression of *PDXP* (Pyridoxal phosphatase) and *PDXK* (Pyridoxine kinase), two isoforms of *PDX* (Pyridoxal 5'-phosphate synthase) — the central structural genes for vitamin B6 biosynthesis—had 3.3- and 10-fold higher expression levels in UV-A leaves, respectively. These upregulated genes belong mainly to the blue module, followed by the yellow and brown modules; thus, the TFs in these modules were used to construct a vitamin regulatory network as described above. Subsequently, 21 TFs, comprising 7 MYB, 4 bZIP, 2 bHLH, 2 GATA, 2C2H2, and 4 other families, were identified as potential regulators for vitamin biosynthesis with the standards of FIMO matching *p* values ≤ 1 e^−4^ and |PCC|≥ 0.8 (Fig. 4C). This result suggests that the MYB family also undertakes critical roles in modulating the biosynthesis of several vitamins induced by UV-A. Phylogenetic analysis revealed that these candidate MYB members displayed high homology to NtMYB305, AdMYB7, SlMYB72, MtWP1, AtMYB113, MlRCP1, and OsRVE1 (Fig. 4D), all of which have been implicated in carotenoid biosynthesis (Jiang et al. 2021; Liang and Li 2023).

### Identification of key transcription factors in regulating sesquiterpenoids biosynthesis upon UV-A radiation

**Fig. 5:**
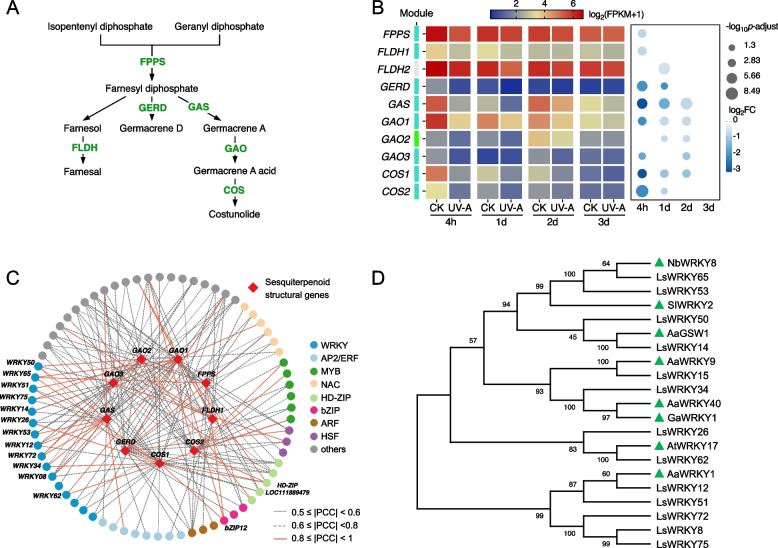
Comprehensive analysis and regulatory network of genes involved in sesquiterpenoid biosynthesis in lettuce under UV-A. **A** Schematic of sesquiterpenoid pathway. **B** Heat map of the genes encoding structural enzymes involved in the sesquiterpenoid biosynthesis pathways. The scaled values of FPKM and Log_2_FC were presented. **C** Regulatory network connecting predicted TFs and key structural genes had potential binding affinity (FIMO match *p*-value ≤ 1e.^−4^) and significant Pearson’s correlation (*p* ≤ 0.01). Expression correlations between TFs and structural genes are shown with line color and type. The full data set is available in Supplementary Table S3. **D** Phylogenetic tree of candidate WRKY proteins with others related to sesquiterpenoid biosynthesis. The known WRKY proteins from other plants were remarked with green triangles. The accession numbers of protein sequences are available in Table S4

Both the metabolome and transcriptome manifested that UV-A radiation negatively affects the accumulation of sesquiterpenoids in lettuce. According to the annotation of the KEGG database, we identified 10 key enzyme-encoding genes involved in the biosynthetic pathway of sesquiterpenoids, which include one *FPS* (farnesyl pyrophosphate synthase), two *FLDH* (farnesol dehydrogenase), one *GERD* (germacrene D synthase), one *GAS* (germacrene A synthase), three *GAO* (germacrene A oxidase), and two *COS* (costunolide synthases) (Fig. 5A, B). Among these, GAS, GAO, and COS undertake the synthesis of the costunolide branch, which serves as the common parent skeleton for the majority of sesquiterpene lactone in lettuce, including lactucin and lactucopicrin (Testone et al., 2019). Notably, all the identified genes presented down-regulated expression levels under UV-A, particularly the genes involved in the costunolide branch (i.e., *GAS*, *GAO*, and *COS*), whose expression levels were 0.12- to 0.57-fold greater in UV-A leaves than in control leaves at 4 h. With the exception of *GAO2*, all the other genes belonged to the turquoise module. The regulatory network of sesquiterpenoids biosynthetic genes and TFs within the turquoise and green modules revealed that the WRKY family was the most plentiful TF family that putatively regulates sesquiterpenoids biosynthesis, which consists of 12 members (Fig. 5C). Inferior to the WRKY family, 3 MYB, 2 NAC, 2 HSF, 2 HD-ZIP, 1 bZIP, and 5 other TFs were also screened out as candidate regulators for sesquiterpenoids biosynthesis (FIMO matching *p* values ≤ 1e^−4^ and |PCC|≥ 0.8). Accessible studies have generally illustrated the direct regulatory role of WRKY TFs in modulating the synthesis of different sesquiterpenoids (Zheng et al. 2023). Hence, the WRKY TFs screened by our network were hired to construct phylogenetic analysis alongside previously reported WRKY members, with the results supporting the potential role of identified WRKY TFs in regulating the synthesis of sesquiterpenoids (Fig. 5D).

### Functional validation of potential transcription factors screened by the regulatory network, taking a sesquiterpenoid biosynthetic gene as a case

**Fig. 6:**
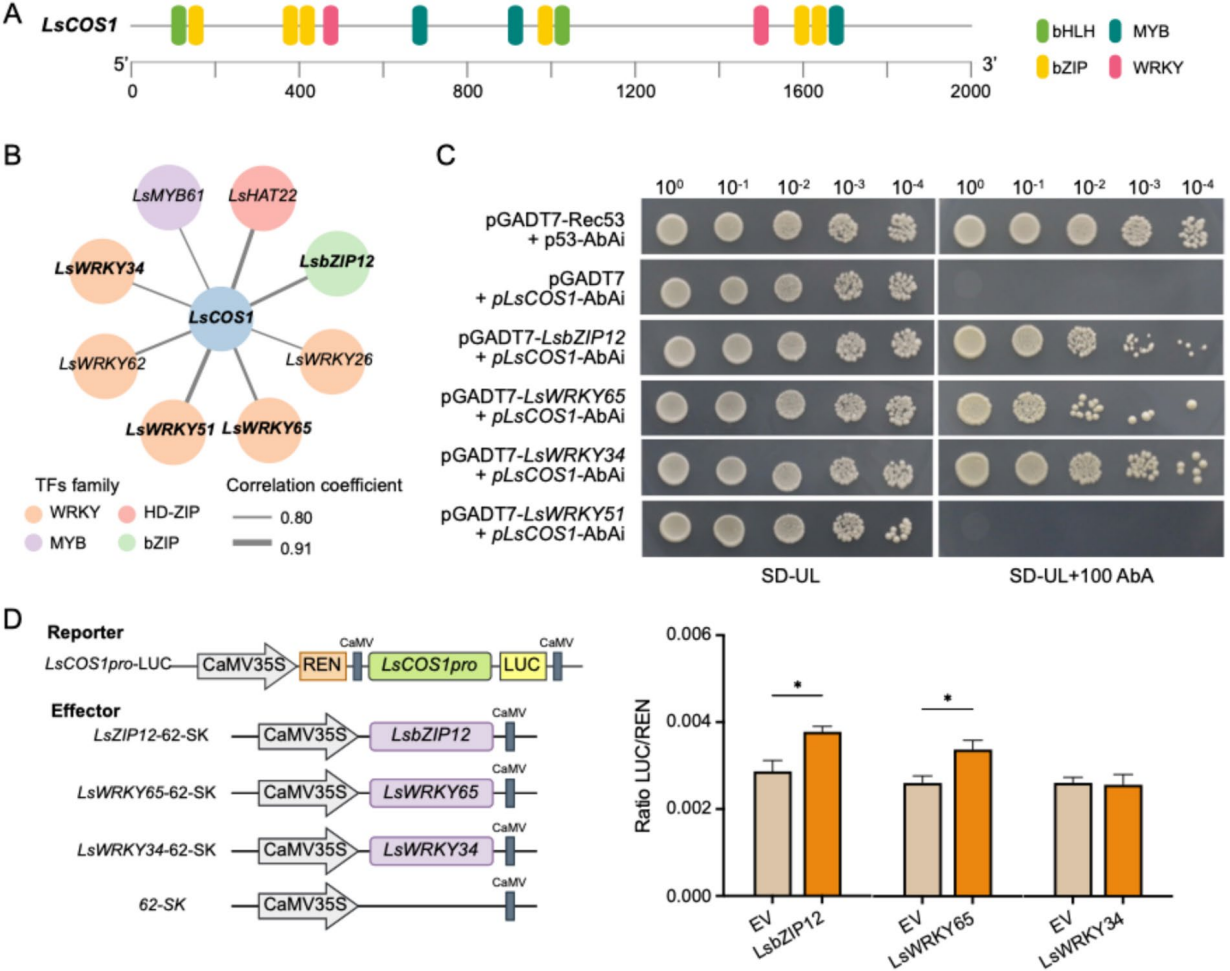
Regulatory effects of hub transcription factors on the promoter of *LsCOS1*. **A** Cis-elements on the promoter of *LsCOS1* identified by Plant CARE. **B** Transcriptional regulatory network of *LsCOS1*. Circle color and line boldness represent the TF family and correlation coefficient, respectively. **C** Yeast one-hybrid analysis of the interaction of hub transcription factors (LsWRKY34, LsWRKY51, LsWRKY65, and LsbZIP12) and *LsCOS1* promoter. **D** Schematic diagram of the constructs utilized in the dual-luciferase assay (left) and the corresponding LUC/REN ratios (right). Data are shown as mean ± SEM (*n* = 3). The asterisks indicate statistical significance (*P* < 0.05) using Student’s t-test

Sesquiterpene lactone is a major subclass of sesquiterpenoids that predominantly exist in the Asteraceae family and contributes great importance concerning medicinal activities and bitterness for lettuce (Yang et al. 2022a). Thus, we further investigated the interaction of candidate TFs with the gene encoding COS, which catalyze the formation of the γ-lactone ring skeleton of most sesquiterpene lactones in lettuce (Testone et al., 2019). Considering the expression levels of *LsCOS* homolog genes (Table S5) and their responses to UV-A radiation (Fig. 5B, Table S6), *LsCOS1* (LOC111890503) was chosen for further investigation. Cis-element analysis revealed putative binding sites of bZIP, WRKY, bHLH, and MYB TFs in the promoter of *LsCOS1* (Fig. 6A). On this basis, in combination with the correlation coefficient and binding affinity between candidate TFs and *LsCOS1* (Fig. 6B), LsWRKY34, LsWRKY51, LsWRKY65, and LsbZIP12 were selected for efficacy authentication. The Y1H assay validated the binding of LsWRKY34, LsWRKY65, and LsbZIP12 to the promoter of *LsCOS1* (Fig. 6C). Therefore, the potential regulatory effects of these three TFs on *LsCOS1* were investigated through dual luciferase reporter assays (Fig. 6D). The results demonstrated that LsWRKY65 and LsbZIP12 activated the expression of *LsCOS1*. Taken together, these results suggest that our metabolic regulatory networks can be used to explore potential regulators for the biosynthesis of bioactive compounds in lettuce.

### Functional recognition of light and phytohormone signaling in UV-A regulation on bioactive compounds

**Fig. 7:**
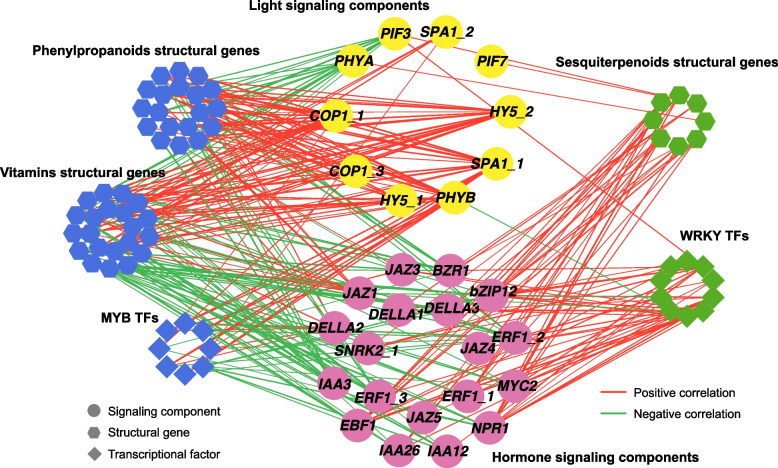
Correlation network of genes encoding light and hormone signaling with biosynthesis genes of bioactive compounds or identified candidate TFs. Circles, hexagons, and diamonds represent signaling genes, structural genes, and transcription factors, respectively. The weight of edges among nodes represents correlation coefficients

WGCNA and KEGG enrichment analysis revealed that the expression patterns of genes related to light signals converged with those responsible for the biosynthesis of UV-A-promoted bioactive compounds, while genes associated with hormone signaling presented consistent expression patterns with sesquiterpenoid biosynthetic genes (Figs. 1F and 2C). Thus, to further elucidate the function of light/hormone signaling in governing bioactive compound synthesis under UV-A, we performed expression correlation analysis of signaling components with preponderant candidate TFs and structural genes (Fig. 7) and constructed a protein–protein interaction (PPI) network of differentially expressed signaling components and candidate TFs (Fig. S3A), as well as regulatory networks of key TFs involved in light/hormone signaling (Fig. S3B, S3C). The PPI network demonstrated the notable protein interactions between light signaling components and hormone signaling components, as well as between several candidate WRKY TFs and hormone signaling components (Fig. S3A). Genes encoding LONG HYPOCOTYL 5 (HY5) and phytochrome interacting factors (PIFs) − pivotal regulators of light signaling − were shown to have high correlations (|PCC|≥ 0.8) and cis binding affinity with several key structural genes responsible for the biosynthesis of phenolic acids (*PAL*, *COMT*), flavonoids (*CHI*, *CHS*, and *F3’H*), pro-vitamin A (*CHYB*, *ZEP*), vitamin E (*VTE3*, *VTE4*), and vitamin B_6_ (*PDX*) (Fig. S3B). In addition, several KEGG annotated hormone-responsive TFs, including ARF, ERF, BZR1, and ABF (bZIP12), were predicted to be repressors of genes responsible for the biosynthesis of phenolic acids (*4CL*, *C4H*, and *CSE*), pro-vitamin A (*ZDS*, *LUT5*, and *VDE*), and vitamin E (*VTE4*), while bZIP12 was also identified as activator of sesquiterpenoids biosynthetic genes (*FPPS*, *GAO*, and *COS*) (Fig. S3C). Moreover, genes encoding identified MYB TFs and enzymes responsible for the biosynthesis of UV-A-promoted bioactive compounds (phenylpropanoids and vitamins) showed strong positive correlations with light signaling-related genes but negative correlations with hormone signaling-related genes, excluding signaling repressors such as PIF3, DELLA, and JAZ (Fig. 7). Whereas identified WRKY TFs and sesquiterpenoid biosynthetic genes only exhibited strong positive correlations with genes encoding hormone signaling components (Fig. 7). These results indicate that light and hormone signaling play distinct roles in mediating UV-A regulation of different bioactive compounds.

## Discussion

### UV-A promotes the accumulation of bioactive compounds with antioxidant or light-absorbing abilities while sacrificing sesquiterpenoid biosynthesis in lettuce

Plant-derived bioactive compounds not only bring health-beneficial effects for humans but also undertake numerous functions in plants. Thus, different bioactive compounds exhibit distinct responses to environmental stimuli. For plants, UV radiation can induce a range of deleterious effects to varying degrees, including damage to DNA, proteins, and lipid membranes, as well as oxidative damage (Neugarta and Schreinera, 2018; Verdaguer et al., 2017). Consequently, to resist UV exposure, plants accumulate many phytochemicals, especially those with antioxidant activity and UV-absorbing properties. Various studies have identified flavonoids and related phenolic compounds as exhibiting significant and widespread responses to UV radiation (Wargent and Jordan 2013). In the present study, UV-A enhanced the overall level of phenolic compounds and flavonoids in lettuce, with more pronounced effects on flavonoids (Fig. 1). This may be related to the fact that the accumulation of flavonoids mainly occurs at intracellular reactive oxygen species production sites (e.g., vacuoles and chloroplasts), which reinforces their antioxidant function (Agati and Tattini 2010, Agati et al., 2012). In addition, most flavonoids exhibit superior absorption ability in the 315–400 nm UV-A range than in the 280–315 nm wavelength range (Cerovic, et al. 2002). Therefore, it can be argued that the accumulation of flavonoids is crucial for plant tolerance to UV-A radiation, which is attributable not only to their antioxidant properties but also to their UV-A absorbing capacity.

In addition to phenylpropanoids, vitamins also possess strong antioxidant potential (Asensi-Fabado and Munné-Bosch, 2010). Our metabolomic and transcriptomic datasets illustrated that UV-A not only definitely improves the accumulation of vitamin B6 and provitamin A but also exhibits potential promotion effects in vitamins E and K (Fig. 1, S1). The free radical scavenging and photoprotection functions of pro-vitamin A, vitamin C, and vitamin E are proverbial (Asensi-Fabado and Munné-Bosch, 2010), and the promotional effects of UV radiation, mainly UV-B, on these vitamins have been reported (Schreiner et al., 2012). A notable windfall of the present study is the vigorous promotional effect of UV-A radiation on the biosynthesis of vitamin B_6_, with up to 20-fold greater levels of 4-pyridoxic acid-O-glucoside in UV-A leave, which has rarely been reported. Research on functional vitamin B6 deficiency in plants has proven that the vitamin B_6_ group also has great antioxidant ability, particularly in response to photooxidative stress, and interacts with tocopherols and carotenoids in chloroplasts (Asensi-Fabado and Munné-Bosch, 2010).

In contrast to phenylpropanoids and vitamins, sesquiterpenoids, particularly sesquiterpene lactones that are exclusively found in Asteraceae plants, were negatively regulated by UV-A (Fig. 1). Although sesquiterpene lactones are the most effective bioactive compounds with therapeutic properties in lettuce, they function mainly as anti-herbivory and anti-microbial substances in plants (Frey et al. 2024). As yet, there appears to be no available research that has confirmed the antioxidant and UV-absorbing ability of sesquiterpene lactones. In addition, our previous study revealed that potential competition for carbon precursors exists between sesquiterpenoids and phenylpropanoids (Zha et al. 2024). Thus, plants may sacrifice ‘useless’ sesquiterpenoids to accumulate phenylpropanoids, which is beneficial for their resistance to UV-A radiation. Intriguingly, sesquiterpene lactones act as a double-edged sword from a dietary perspective, as they are accompanied by bitterness (Anilakumar et al., 2017; Frey et al. 2024). So, on another note, UV-A can be considered effective in improving the sensory quality of lettuce. In general, UV-A has positive effects on the accumulation of bioactive compounds with antioxidant or light-absorbing abilities, mainly including phenylic acids, flavonoids, provitamin A, vitamin E, vitamin K_1_, and vitamin B_6_, but represses the biosynthesis of sesquiterpenoids.

### MYB TFs act as master regulators in activating phenylpropanoid and vitamin biosynthesis under UV-A

Transcriptional regulation is the major mechanism governing the biosynthesis of defensive compounds under environmental stimulations (Colinas and Goossens 2018). In recent decades, MYB TFs have been extensively reported to engage in the transcriptional regulation of many phytochemicals, particularly phenylpropanoids and carotenoids (Cao et al., 2020; Cao et al., 2024; Li et al. 2022). Here, WGCNA and regulatory network analysis indicated that MYB family TFs were the most abundant TFs, exhibiting strong coexpression and binding affinity with upregulated structural genes responsible for the biosynthesis of phenylpropanoids and vitamins. These findings suggest a master regulatory role for MYB TFs in enhancing these bioactive compounds under UV-A radiation in lettuce. Among the identified MYBs, five members (LsMYB4/8/111/75/114) belong to the R2R3-MYB family (Fig. 3), a subfamily that is widely dismissed as crucial regulators governing the biosynthesis of phenylpropanoids (Cao et al., 2024). Among them, LsMYB4 and LsMYB111 exhibited specific regulatory effects on the genes responsible for the flavonoid biosynthesis branch and clustered closely with AtMYB111, AtMYB12, and AtMYB11, celebrity MYB members known for particularly activating the genes encoding CHI, CHS, FLS, and F3H (Pratyusha and Sarada 2022). Meanwhile, LsMYB75 and LsMYB114 share high homology with AtMYB113, AtMYB114, AtMYB75, and AtMYB90, which are specifically known for their role in the upregulation of phenylpropanoids synthesis (Cao et al. 2020). In addition to phenylpropanoids, LsMYB8 and LsMYB75 have also emerged as credible transcriptional activators in carotenoid synthesis by directly targeting *LsZISO* and *LsLCYB*, respectively, substantiated by phylogenetic evolution analysis (Fig. 4). As shown in the phylogenetic tree, LsMYB8 and LsMYB75 clustered close to SlMYB72 and MtWP1, the former directly activated the expression of *SlPSY* and *SlZISO* in *Solanum lycopersicum* (Wu et al. 2020), and the latter regulated carotenoid biosynthesis in *Medicago truncatula* by modulating the expression of *MtLCYE* and *MtLCYB* (Meng et al. 2019).

Beyond R2R3 type MYBs, MYB-related MYBs (R1-MYB) also participate in the regulation of phenylpropanoid and carotenoid accumulation, particularly in terms of light regulation, as MYB-related TFs are components of central circadian oscillators (Pérez-Llorca and Müller, 2024). In this study, several MYB-related type MYBs, LsMYB1R1, LsRVE8, and LsRVE1, whose corresponding closest homologous protein in Arabidopsis is AtMYBD, AtRVE8, and AtRVE1, respectively, were also screened out implicated in upregulation of phenylpropanoids and vitamins (Figs. 3 and 4). Align with our results, AtMYBD, AtRVE8, and CstMYB1R1 have been authenticated as regulators of flavonoid and anthocyanin biosynthesis (Bhat et al., 2024; Nguyen et al., 2015; Pérez-García et al., 2015). Furthermore, the orthologs of plant RVE1s have been reported to positively regulate carotenoid levels in rice (Jeong et al., 2022) and activate chlorophyll biosynthesis in Camellia sinensis (Liu et al. 2023a). Concomitantly, a recent study by Liang et al., (2023), deduced that LsMYB1R1, LsMYB8, and LsMYB75 participate in the light regulation of flavonoid biosynthesis in lettuce through comparative transcriptomic analysis, backing up our results positively. In conjunction with the well-documented roles of MYB TFs in the modulation of phenylpropanoids and carotenoids, our datasets offer novel insights into the involvement of MYBs in governing the biosynthesis of vitamin E, K_1_, and B_6_. These findings cement MYB TFs as promising candidates for delving into UV-A-induced bioactive compound synthesis mechanisms.

### WRKY TFs act as master regulators in repressing sesquiterpenoid biosynthesis under UV-A

Comparatively, much less is known about the transcriptional regulatory mechanism governing sesquiterpenoid synthesis, as most sesquiterpenoids are species-specific. Nonetheless, limited results have indicated that WRKY TFs emerge as predominant regulators of sesquiterpenoid biosynthesis (Schluttenhofer and Yuan 2015; Zheng et al. 2023). Within this study, among the 27 identified candidate TFs involved in the regulation of sesquiterpenoid biosynthesis, WRKY constituted the largest family, which contained 12 members (Fig. 5C). This finding provides valuable evidence for the critical roles of WRKY TFs in sesquiterpenoid biosynthesis. Up to now, Artemisinin has been the most intensively studied sesquiterpene lactone compound in terms of biosynthesis-related transcriptional regulatory mechanisms, which is attributed to its highly effective antimalarial activity (Zheng et al. 2023). Several WRKY members have been identified to regulate artemisinin biosynthesis by activating the expression of artemisinin biosynthetic pathway genes, including AaWRKY1, AaWRKY9, AaWRKY14, and AaWRKY17 (Zheng et al. 2023). Other than in Artemisia, GaWRKY1 was characterized as a regulator of the sesquiterpene cyclase ((1)-d-cadinene synthase) in cotton (Xu et al., 2004). Meanwhile, in *Saussurea lappa*, SlWRKY2 positively interacted with the gene encoding diphosphomevalonate decarboxylase (Thakur et al., 2021). Concordantly, WRKY TFs identified in our study as regulators of sesquiterpenoid biosynthesis presented high homology to these reported WRKYs (Fig. 5D). The principal sesquiterpene lactones in lettuce (lactucin, lactucopicrin, and their derivatives) are derived from costunolide, which is synthesized through FPPS, GAS, GAO, and COS (Testone et al., 2019). Regrettably, unlike artemisinin, authenticated transcriptional regulation of costunolide biosynthesis has not yet been reported; present studies only predicted several potential candidate TFs through transcriptomic-based coexpression analysis, including WRKY family members (Testone et al., 2019; Thakur et al., 2020). In *Saussurea lappa*, the *SlGAS1* gene was found to be coexpressed with the WRKY TF family (Thakur et al., 2020). In addition, the expression trends of genes encoding GAS, GAO, and COS in *Cichorium endivia* were inversely correlated with sesquiterpene lactones (lactucin and lactucopicrin) abundance, as well as a WRKY TF (Ce_contig 86,458), which is homologous to LsWRKY75 (XP_023737392.1) (Testone et al., 2019). Moreover, promoter analysis verified that the WRKY binding domain particularly coexists in the promoters of genes involved in the costunolide branch in lettuce (Fig. S5), as are the *COS1* gene in *Saussurea lappa* (Thakur et al., 2020) and the *GAS* gene in *Cichorium endivia* (Testone et al., 2019). Further experiments also confirmed that LsWRKY65 can bind to the promoter of *LsCOS1* and activate its expression (Fig. 6). Collectively, these results provide compelling evidence highlighting the critical function of WRKT TFs in modulating the biosynthesis of sesquiterpene lactones.

### Distinct roles of light and hormone signaling in the regulation of UV-A on different bioactive compounds

It is incontrovertible that light signaling plays a paramount role in the UV-A-mediated transcriptional regulation of bioactive compounds. In parallel, it has been extensively found that the activities of various plant hormones can be influenced to varying degrees by light intensity and spectrum (Cao et al., 2024; Sun et al. 2024) and multilevel interactions between the light signaling pathway and hormone signaling pathways under abiotic and biotic stimuli (Wang et al., 2019; Qian et al. 2020). Hub positive and negative regulatory factors of the light signaling cascade, i.e., HY5 and PIFs, were identified as key integrators that link light signals to phytohormone signaling (Lau and Deng 2010). Here, the protein interaction network of the differentially expressed light and hormone signaling components suggested that strong interactions occurred between the light and hormone signaling pathways through PIF3 under UV-A light (Fig. S3A). In addition, we found that light and hormone signal transduction processes exhibited contrary responses to UV-A radiation (Fig. 1F) and were separately clustered with the metabolic processes of different bioactive compounds (Fig. 2C). Therefore, we speculate that light and hormone signals play distinct roles in UV-A-mediated transcriptional regulation of different bioactive compounds.

For bioactive compounds with light-absorbing ability, such as flavonoids and carotenoids, the dominant role of light signal in transcriptional regulation governing the biosynthesis of these compounds has been extensively proven (Liu et al. 2023b). HY5—the pivotal regulator of the light-mediated transcriptional regulatory network—not only can directly regulate the expression of the structural genes involved in the biosynthesis of phenolic acids (*PAL*), flavonoids (*CHS*, *CHI*, *FLS*, *F3H*, and *F3’H*), carotenoid (*PSY*, *Z-IS*O, *CrtISO*1, *LCY*B, *LCYE*, and *VDE*) (Gangappa and Botto 2016; Liu et al. 2023b; Wang et al. 2021) but also inducing transcriptional activators of these genes, such as abovementioned AtMYB11, AtMYB12, AtMYB111, AtMYB75, and AtMYBD (Gangappa and Botto 2016; Pech et al., 2022). Furthermore, PIFs are generally reported to act antagonistically with HY5 and negatively regulate the biosynthesis of phenylpropanoids and carotenoids in direct and/or cascade transcriptional regulation mode (Liu et al. 2023b). Concordantly, genes encoding HY5 and PIF3 identified in our study exhibited high correlations (|PCC|≥ 0.8) and cis-binding affinity with key structural genes involved in phenolic acids, flavonoids, provitamin A, and vitamin B6 biosynthesis (Fig. S3B), as well as strong correlations with identified candidate MYB regulators (Fig. 7), supporting function similarities of LsHY5/LsPIFs to prior studies. Apart from light signaling, light-induced biosynthesis of various phytochemicals is also modulated by hormones (Brini et al., 2022). It has been reported that UV-B radiation can inhibit several hormone-signaling processes, which alleviates the inhibitory effects of hormone-responsive factors on structural genes or activators responsible for flavonoid biosynthesis (Song et al., 2022). In our context, multiple hormones signaling processes were suppressed by UV-A (Figure S4), and several hormone-responsive TFs, including ARF, ERF, BZR1, and ABF (bZIP12), were identified as transcriptional repressors of the structural genes involved in phenolic acids, provitamin A, and vitamin E biosynthesis (Figure S3C). Accordingly, we propose that restriction of the hormone signal transduction induced by UV-A leads to the degradation of hormone-responsive negative regulators, thereby contributing to the accumulation of UV-A-induced bioactive compounds.

Although light has notable impacts on the accumulation of sesquiterpenoids (Fonseca et al. 2006; Fu et al. 2021), no experimental evidence has yet demonstrated that light signal regulators can independently and directly regulate sesquiterpenoid biosynthesis genes (Liu et al., 2023c; He et al. 2024). In the context of *Artemisia annua*, HY5 or COP1 administers artemisinin biosynthesis by working either individually (Hao et al. 2019) or with other interactors (Liu et al., 2023c; He et al. 2024; Fu et al. 2021) to regulate downstream TFs, which can directly bind to the promoters of sesquiterpene lactone biosynthesis genes. Additionally, available results also have suggested that the integration of light and phytohormone signaling is involved in the regulation of sesquiterpene lactone biosynthesis (Zheng et al. 2023; Liu et al., 2023c; Fu et al. 2021). Meanwhile, several hormone-responsive factors, such as MYC2, ABF, and TGA, have been confirmed to either directly target or indirectly modulate the structural genes and TFs involved in sesquiterpene lactone biosynthesis (Zhang et al. 2015; Zheng et al. 2023; Xu et al., 2017; Shen et al. 2016). In this study, three-way significant positive correlations among the expression levels of hormone signaling genes, sesquiterpenoid biosynthetic genes, and identified candidate WRKY TFs (Figs. 5C and 7), as well as notable protein interactions between WRKY TFs and hormone signaling components (Figure S3A), were observed. Furthermore, WRKY TF was considered a critical link between phytohormones and metabolic processes in plants (Jiang and Yu 2015). In addition, the abscisic acid-responsive factor (bZIP12) was identified as a potential activator of *FPPS*, *GAO1*, and *COS1*, and its interaction efficacy with *COS1* was verified by Y1H and dual luciferase reporter assay (Fig. 6). Conversely, neither WRKY TFs nor sesquiterpenoid biosynthetic genes exhibited notable correlations with light signaling components (Fig. 7). Taken together, these findings provide substantial evidence that hormone signaling dominates a more immediate role in the cascade transcriptional regulation of UV-A on sesquiterpenoid biosynthesis than light signaling. The inhibitory effect of UV-A on sesquiterpenoid biosynthesis appears to be the knock-on effect of attenuated hormone signal transduction, and the WRKY TF has great potential in mediating this process.

**Fig. 8:**
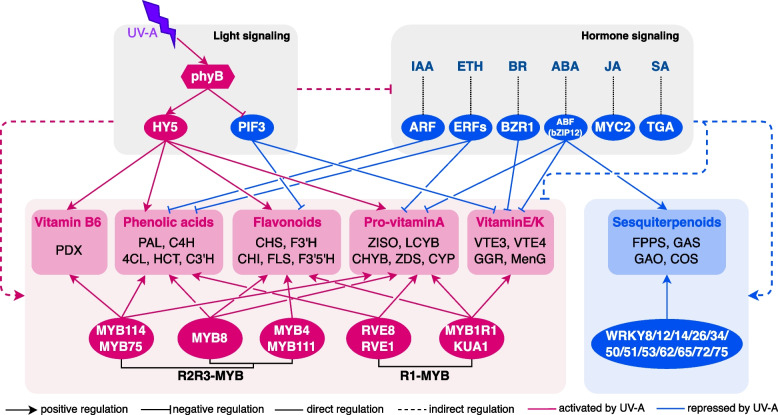
The schematic diagram represents the regulatory mechanisms that underlie UV-A regulation on the biosynthesis of bioactive compounds in lettuce. Ellipses represent transcription factors; rectangles represent target structural genes. Red and blue backgrounds indicate that gene expression or compound levels were up- and down-regulated by UV-A, respectively

In summary, our study provides a comprehensive overview of the accumulation features of the major bioactive compounds in lettuce upon UV-A radiation. Additionally, we have delivered an integrated transcriptional regulatory network involving light/hormone signaling components, transcription factors, and target structural genes, which elucidates the regulatory mechanism of UV-A on the biosynthesis of bioactive compounds (Fig. 8). Furthermore, the reliability and referenceability of our findings were demonstrated through integrated bioinformatics analyses and functional genomics assays. Consequently, this research contributes significant insights into identifying novel transcription factors that modulate the transcriptional regulation of key bioactive compounds, thereby providing a foundation for enhancing the bioactivity value of lettuce and other vegetable crops.

## Materials and methods

### Plant materials, growth conditions, and sample harvest

A green leafy cultivar of lettuce (Lactuca sativa L. cv. ‘Yidali’) was grown in an environment-controlled plant factory at 23/20 °C (light/dark) and a relative humidity of 65%. Seeds were germinated in rock wool under white LED light (170 μmol·m^−2^·s^−1^, 16 h light/8 h dark) for two weeks; after that, the plants were kept in darkness for 48 h. Subsequently, uniform plants were randomly divided into two groups and treated with 170 μmol·m^−2^·s^−1^ red light (control, CK) or 170 μmol·m^−2^·s^−1^ red light plus 30 μmol·m^−2^·s^−1^ UV-A light (UV-A), respectively. The photoperiod during the experiment was 16 h light/8 h dark. The red and UV-A light were provided by LED sources with peak wavelengths of 660 and 385 nm, respectively. A spectroradiometer (Avaspec-2048CL, Avantes, Netherlands) was used to monitor and maintain the set light intensity of each light spectrum at the height of the plant canopy. Leaf samples from each treatment were collected after treatment for 4 h, 1 day, 2 days, or 3 days and frozen in liquid immediately. The newest three fully expanded leaves from five plants were mixed into one biological sample, with each treatment comprising three biological replicates. Samples collected at 1 d, 2 d, and 3 d were obtained at the 10th hour of the light period. Given that transcriptional responses often occur faster than metabolic changes do, the samples collected at 1 d, 2 d, and 3 d were utilized for both transcriptomic and metabolomic analyses, whereas those collected at 4 h were used only for transcriptomic analyses. The plants were irrigated daily with Hoagland nutrient solution (pH 5.8, EC 1.6 dS·m^−1^).

### Metabolomic profiling

A liquid chromatography − mass spectrometry (LC − MS)-based wide targeted metabolomic method was applied for metabolomic profiling, as the majority of bioactive compounds contained in lettuce are polar and can be extracted via aqueous alcoholic solvents (Yang et al. 2022a). Lyophilized powder samples (100 mg) were dissolved in 70% methanol (1.2 mL) and extracted for 12 h at 4 °C. After centrifugation, the supernatant was filtered through a 0.22 μm filter and then underwent UPLC analysis via a Nexera X2 system (Shimadzu, Japan) coupled with ESI–MS/MS (Applied Biosystems 6500 Q TRAP, America). Metabolite fractions were identified through a triple quadrupole (QQQ)-linear ion trap mass spectrometer equipped with an ESI Turbo Ion-Spray interface operating in both positive (5500 V) and negative (− 4500 V) ion modes. The MRM mode was used during the QQQ scans. Specific sets of MRM transitions were identified on the basis of the eluted metabolites within each period. Data acquisition and validation were carried out via Analyst 1.6.3 (AB Sciex). To determine the differences in the relative contents of metabolites between treatments, *p* values ≤ 0.05 and VIP ≥ 1 were determined as the thresholds of univariate statistical analysis and multivariate statistical analysis, respectively, which were obtained from paired t-tests and the OPLS-DA model.

### Transcriptomic profiling

Total RNA extraction and purification were conducted via TRIzol Reagent and DNase I (TaKaRa). The integrity and quantity of total RNA were detected with an Agilent 2100 bioanalyzer (Agilent, USA) and an ND-2000 (Thermo, USA). The RNA-seq library was subsequently prepared via the TruSeqTM RNA Sample Preparation Kit (Illumina, USA) and then subjected to sequencing on an Illumina NovaSeq 6000 sequencing platform with a 2 × 150 bp read length (Majorbio, China). The raw paired-end reads underwent trimming and quality control by fastp (Chen et al. 2018). The resulting clean reads were subsequently aligned to the Lactuca sativa genome (https://www.ncbi.nlm.nih.gov/datasets/genome/GCF_002870075.4/) in orientation mode via HISAT2 software (Kim et al. 2015). The mapped reads from each sample were then assembled via StringTie via a reference-based methodology (Pertea et al. 2015). The gene expression level was quantified via the fragments per kilobase per million reads (FPKM) method, while gene abundances were assessed via RSEM (Li and Dewey, 2011). To identify differential expression between the two comparison combinations, DESeq2 was employed (Love et al. 2014), and Benjamini and Hochberg’s method was applied to adjust the resulting *p* values, thus controlling the false discovery rate (FDR). Next, the KEGG database and KOBAS software were used to annotate functional genes and analyze KEGG pathway enrichment.

### Weighted gene coexpression network analysis (WGCNA)

After discarding genes with average FPKM < 1, the union of DEGs (*p*-adjust ≤ 0.05 and |log_2_-fold change|≥ 0.58, i.e., fold change (FC) ≥ 1.5 or ≤ 0.67) at each time point was imported to construct the coexpression modules by the R package ‘WGCNA’ using the automatic network construction function (block-wise modules). Specifically, the power estimate value, min module size, merge cut height, and min KME to stay were set to 13, 30, 0.25 and 0.3, respectively. After module recognition, the correlation coefficients between modules and traits were calculated.

### Construction of the bioactive compound biosynthesis regulatory network and protein–protein interaction (PPI) analysis

The Plant Transcription Factor Database (PlantTFDB v4.0) was used to predict transcription factors (TFs) and obtain binding motifs. The sequences of the structural gene promoter regions (2000 bp upstream of the initiation codon) were extracted by TBtools software (Chen et al. 2023). Then, the binding ability to the cis-elements was predicted by FIMO under the condition of a *p* value ≤ 1 e^−4^ (https://meme-suite.org/meme/tools/fimo). The transcriptional regulatory networks were created by integrating the availability of cis-element binding sites present in the promoter regions of structural genes alongside the Pearson correlation coefficient (PCC ≥ 0.5, *p* ≤ 0.01) between TFs and structural genes in the modules with high correlation. These networks were visualized via Cytoscape (version 3.9.1, USA). PPI analysis was performed by using the STRING database (https://string-db.org/) and then further visualized via Cytoscape.

### Phylogenetic tree and promoter cis-acting element analysis

The associated amino acid sequence alignments were conducted utilizing the MUSCLE algorithm implemented in MEGA7 (Kumar et al. 2018). Molecular phylogenetic analysis was executed using an NJ matrix-based model, with bootstrap values calculated from 1000 replicates. The neighbor-joining tree was generated in MEGA7. Plant CARE (https://bioinformatics.psb.ugent.be/webtools/plantcare/html/) was employed for the prediction of cis-acting elements within the promoter region (2000 bp upstream of the initiation codon) of candidate genes. The predictive data were subsequently integrated into TBtools software for mapping.

### RT‒qPCR

The expression levels of 20 vital genes in lettuce were determined via RT‒qPCR to confirm the reliability of sequencing-based transcriptome profiling. RT‒qPCR was conducted with a CFX384 Touch Real-Time PCR Detection System using a Talent qPCR PreMix (SYBR Green) Kit (TIANGEN, China). The gene expression levels were calculated using the 2^−ΔΔCT^ method. 18S rRNA was used as the housekeeping gene. The sequences of primers used for the qPCR and expression levels of the selected genes are listed in Table S6. Data are shown as fold change of UV-A treatment relative to the control on 4h.

### Y1H assay

The full-length CDS sequences of *LsWRKY34*, *LsWRKY51*, *LsWRKY65*, and *LsbZIP12* were individually inserted into pGADT7 prey vector and obtained the pGADT7-LsbZIP12, pGADT7-LsWRKY34, pGADT7-LsWRKY51, and pGADT7-LsWRKY65 plasmids. The promoter fragment (2000 bp upstream of the ATG codon) of LsCOS1 was recombinant into pAbAi bait vector and obtained pLsCOS1-AbAi plasmid. The pLsCOS1-pAbAi vector and positive control vector p53-AbAi were linearized by BbsI-HF (New England Biolabs, Massachusetts, USA) and transformed into the Y1H yeast competent cells, then coated separately on SD/-Ura plates with different gradients of AbA to determine the minimum AbA inhibitory concentration. Subsequently, pLsCOS1-pAbAi was transfected into Y1H yeast competent cells with pGADT7-Rec53 (positive control), pGADT7 (negative control), pGADT7-LsbZIP12, pGADT7-LsWRKY34, pGADT7-LsWRKY51, and pGADT7-LsWRKY65 plasmid, respectively, and then coated on the SD/-Ura/-Leu plates without AbA and SD/-Ura/-Leu plates with the lowest AbA inhibition concentration (100 ng/mL) to verify the interaction relationship between them.

### Dual-luciferase reporter assay

The full-length CDS sequences of *LsWRKY34*, *LsWRKY65*, and *LsbZIP12* were individually incorporated into the pGreenII-62-SK vector to generate the effectors, while the 2000 bp promoter sequence of *LsCOS1* was recombinant cloned into pGreenII 0800- LUC vector to generate the reporter. After that, effectors and *LsCOS1*pro-LUC double reporter were transformed into Agrobacterium tumefaciens strain GV3101 (pSoup-p19). Each effector and *LsCOS1*pro-LUC reporter were co-infiltrated into N. benthamiana leaves at a 2:1 volume ratio. The empty pGreenII-62-SK vector served as the control. Leave samples were collected after 48h dark treatment and detected by the Dual-Luciferase Reporter Assay System (E1910, Promega, USA) to assess luciferase activity and obtain LUC/REN ratio. Three independent transformations were performed for each assay.

## Supplementary Information

Additional file1: Fig. S1. Phenotype and photosynthetic pigment contents of lettuce grown under UV-A. Fig. S2. Module−trait correlations of WGCNA analysis. Fig. S3. Regulation role of light and phytohormone signaling in UV-A regulation on bioactive compounds. Fig. S4. Enrichment of DEGs on the ‘plant hormone signal transduction’ pathway. Fig. S5. Cis-elements on the promoter of sesquiterpenoid structural genes.

Additional file 2: Table S1. Detailed metabolomics data for bioactive compounds in lettuce. Table S2. Detailed results of WGCNA analysis. Table S3. Detailed data of transcriptional regulatory networks for bioactive compounds. Table S4. Accession number of proteins applied for phylogenetic tree analysis. Table S5. FPKM values of LsCOS homology genes. Table S6. Primers and relative expression levels of genes that verified using qRT-PCR.

## Data Availability

All data supporting the results of this study can be found within the manuscript and its supplementary materials.

## Abbreviations

4CL: 4-Coumarate CoA ligase
C3′H: P‐coumaroyl shikimate 3′ hydroxylase
C4H: Cinnamate-4-hydroxylase
CCoAMT: Caffeoyl CoA 3‐O‐methyltransferase
CCR: Cinnamoyl-CoA reductase
CHI: Chalcone isomerase
CHS: Chalcone synthese
CHYB: β-carotene hydroxylase
COMT: Caffeic acid 3-O-methyltransferase
COS: Costunolide synthase
crtISO: Prolycopene isomerase
CSE: Caffeoyl shikimate esterase
F3′H: Flavonoid 3′‐hydroxylase
F3H: Flavanone 3-hydroxylase
F5H: Ferulate 5-hydroxylase
FLDH: Farnesol dehydrogenase
FLS: Flavonol synthese
FPPS: Farnesyl pyrophosphate synthase
GAO: Germacrene A oxidase
GERD: Germacrene D synthase
GGPPS: Geranylgeranyl pyrophosphate synthase
GGR: Geranylgeranyl diphosphate reductase
HCT: Shikimate O-hydroxycinnamoyl transferase
HY5: Long hypocotyl 5
KEGG: Kyoto Encyclopedia of Genes and Genomes
LCYB: Lycopene beta cyclase
LCYE: Lycopene epsilon cyclase
LUT1: Carotene epsilon-monooxygenase
LUT5: β-ring hydroxylase
MenA: 2-Carboxy-1,4-naphthoquinone phytyl transferase
MenG: 2-Phytyl-1,4-beta-naphthoquinone methyltransferase
NDC1: Demethylphylloquinone reductase
PAL: Phenylalanine ammonia-lyase
PDS: 15-Cis-phytoene desaturase
PDX: Pyridoxal 5'-phosphate synthase
PDXK: Pyridoxine kinase
PDXP: Pyridoxal phosphatase
PIF: Phytochrome interacting factors
PLR: Pyridoxal reductase
PPOX: Pyridoxine/pyridoxamine 5'-phosphate oxidase
PSY: Phytoene synthase
VDE: Violaxanthin de-epoxidase
VTE1: Tocopherol cyclase
VTE2: Homogentisate phytyl transferase
VTE3: Methylphytylbenzoquinol methyltransferase
VTE4: Tocopherol O-methyltransferase
ZDS: Zeta-carotene desaturase
ZEP: Zeaxanthin epoxidase
ZISO: 15-Cis-zeta-carotene isomerase
