# Supplementary material for: Integrative metabolome and genome-wide transcriptome analyses reveal the regulatory network for bioactive compound biosynthesis in lettuce upon UV-A radiation
Source: Mol Hortic. 2025 Aug 5;5:45. doi: 10.1186/s43897-025-00163-1 (PMC12323076; doi:10.1186/s43897-025-00163-1)
Supplement: Supplementary file 1 — Additional file1: Fig. S1. Phenotype and photosynthetic pigment contents of lettuce grown under UV-A. Fig. S2. Module−trait correlations of WGCNA analysis. Fig. S3. Regulation role of light and phytohormone signaling in UV-A regulation on bioactive compounds. Fig. S4. Enrichment of DEGs on the ‘plant hormone signal transduction’ pathway. Fig. S5. Cis-elements on the promoter of sesquiterpenoid structural genes. [file 43897_2025_163_MOESM1_ESM.pdf]

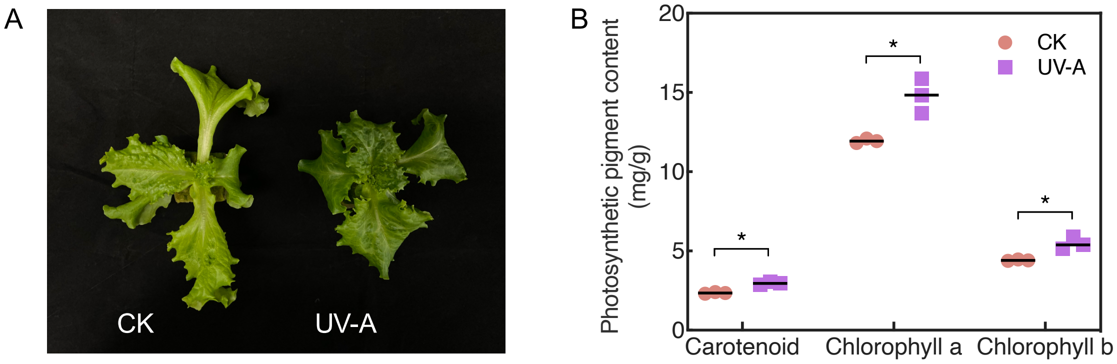

Fig. S1 Phenotype (A) and photosynthetic pigment contents (B) of lettuce grown under control (CK) and UV-A on 3d.

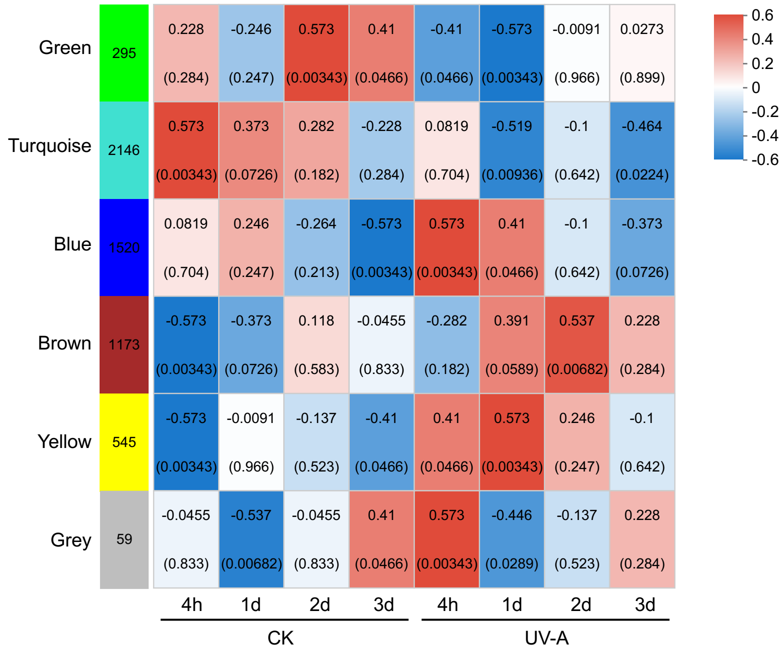

Fig. S2 Correlation between module and trait of WGCNA analysis.

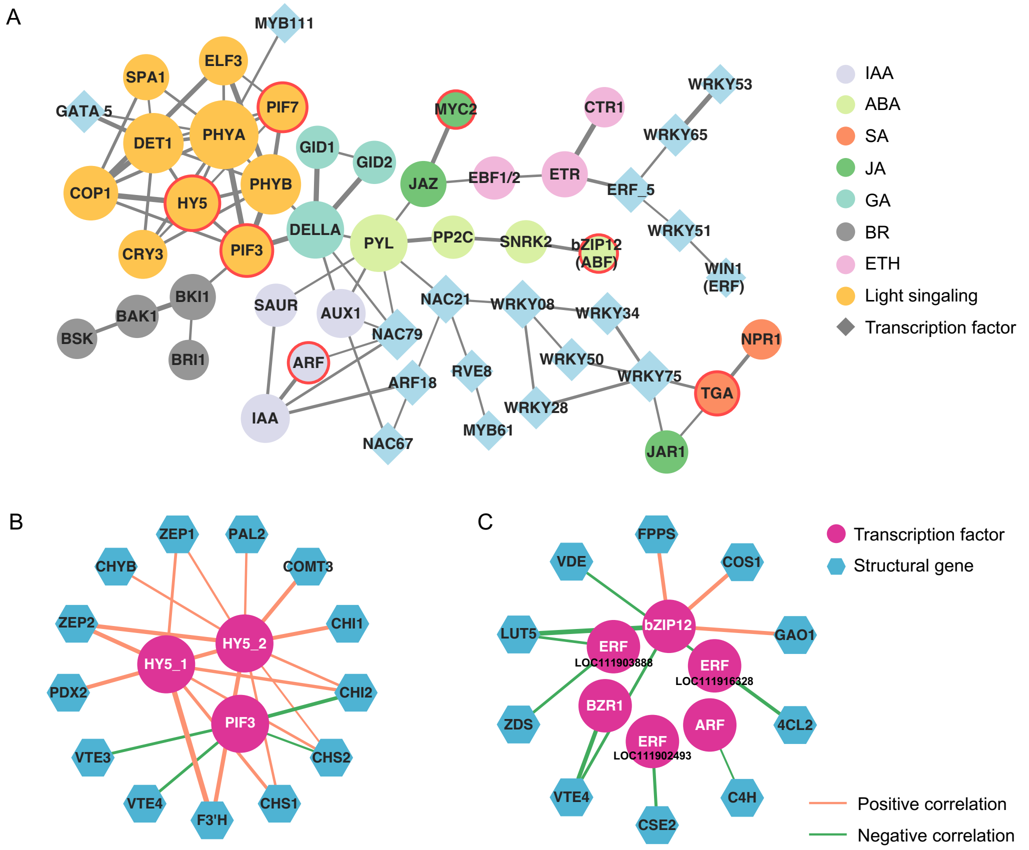

Fig. S3 Regulation role of light and phytohormone signaling in UV-A regulation on bioactive compounds. (A) Protein-protein interaction (PPI) network of differentially expressed signaling components and candidate TFs. Node size indicates connectivity, and line thickness indicates combined score based on STRING database. (B) Regulatory network of key TFs involved in light signaling. (C) Regulatory network of key TFs involved in hormone signaling.

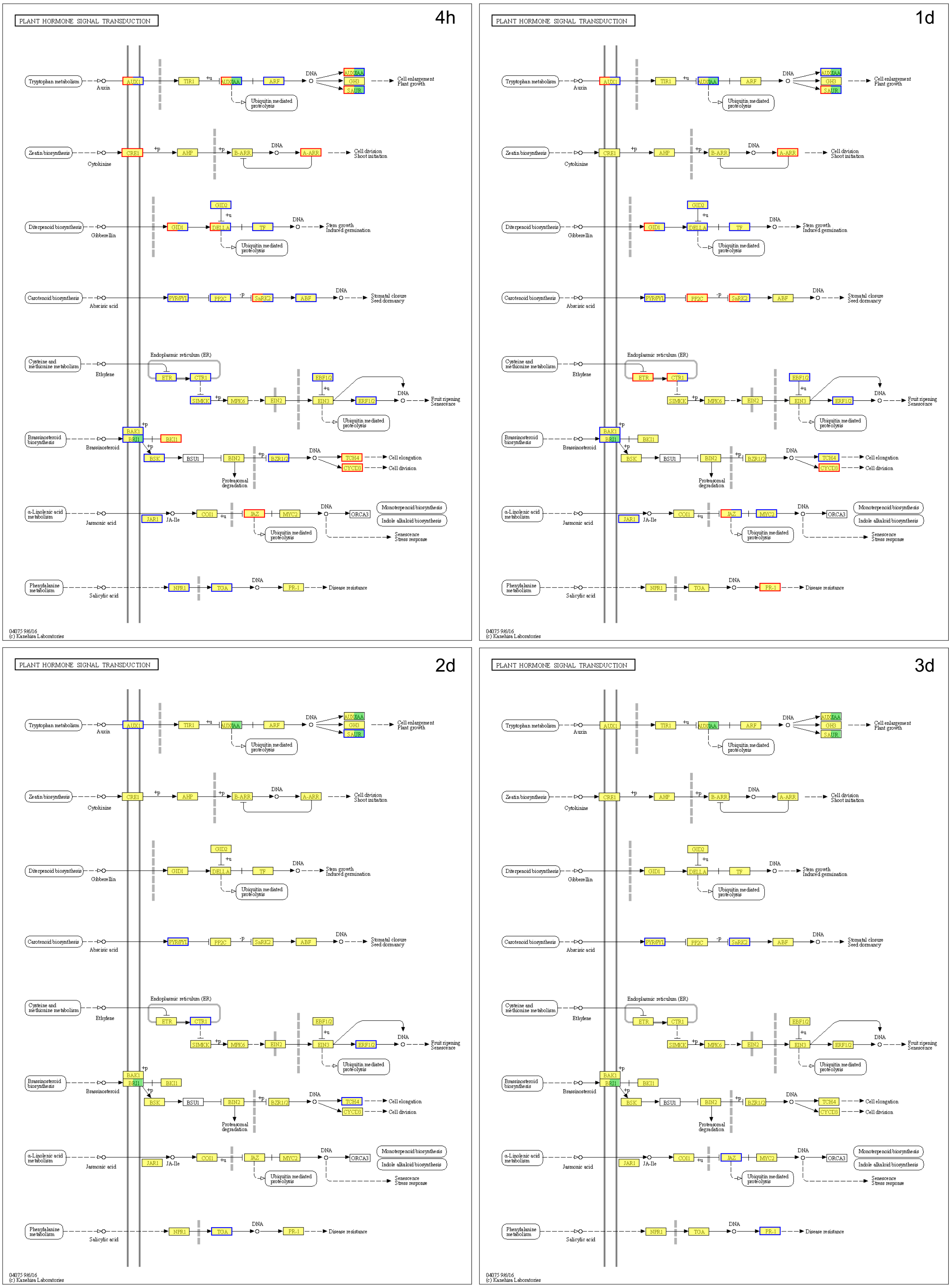

Fig. S4 Enrichment of DEGs on the ‘plant hormone signal transduction’ pathway. Red, blue, and red+blue border color indicate genes encoding the corresponding protein were upregulated, downregulated, and up+downregulated by UV-A, respectively.

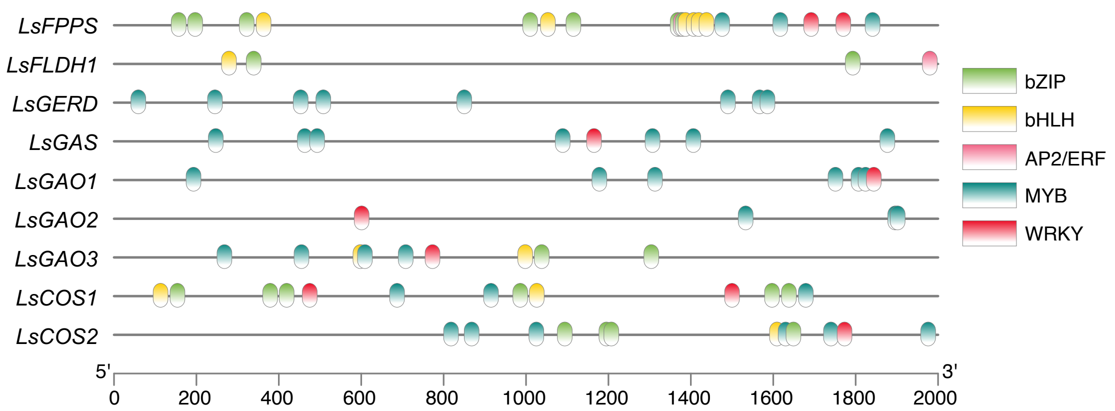

Fig. S5 Cis-elements on the promoter of sesquiterpenoid structural genes. Cis-elements were identified by Plant CARE and visualised via TBtools.
